# Do Programs for Runaway and Homeless Youth Work? A Qualitative Exploration From the Perspectives of Youth Clients in Diverse Settings

**DOI:** 10.3389/fpubh.2018.00112

**Published:** 2018-04-19

**Authors:** Marya Gwadz, Robert M. Freeman, Alexandra H. Kutnick, Elizabeth Silverman, Amanda S. Ritchie, Charles M. Cleland, Noelle R. Leonard, Aradhana Srinagesh, Jamie Powlovich, James Bolas

**Affiliations:** ^1^Center for Drug Use and HIV Research, Rory Meyers College of Nursing, New York University, New York, NY, United States; ^2^Coalition for Homeless Youth, New York, NY, United States

**Keywords:** runaway behavior, homeless youth, positive youth development, qualitative research, effectiveness, programs, organizations, Youth Program Quality Assessment

## Abstract

Runaway and homeless youth (RHY) comprise a large population of young people who reside outside the control and protection of parents and guardians and who experience numerous traumas and risk factors, but few buffering resources. Specialized settings have developed to serve RHY, but little is known about their effects. The present cross-sectional qualitative descriptive study, grounded in the positive youth development approach and the Youth Program Quality Assessment model, addressed this gap in the literature. From a larger sample of 29 RHY-specific settings across New York State, RHY ages 16–21 from 11 settings were purposively sampled for semi-structured in-depth interviews on their transitions into homelessness, experiences with settings, and unmet needs (*N* = 37 RHY). Data were analyzed with a theory-driven and inductive systematic content analysis approach. Half of participants (54%) were female; almost half (49%) identified as non-heterosexual; and 42% were African American/Black, 31% were Latino/Hispanic, and 28% were White/other. Results indicated that because RHY are a uniquely challenged population, distrustful of service settings and professional adults and skilled at surviving independently, the population-tailored approaches found in RHY-specific settings are vital to settings’ abilities to effectively engage and serve RHY. We found the following four major themes regarding the positive effects of settings: (1) engaging with an RHY setting was emotionally challenging and frightening, and thus the experiences of safety and services tailored to RHY needs were critical; (2) instrumental support from staff was vital and most effective when received in a context of emotional support; (3) RHY were skilled at survival on the streets, but benefited from socialization into more traditional systems to foster future independent living; and (4) follow-through and aftercare were needed as RHY transitioned out of services. With respect to gaps in settings, RHY highlighted the following: (1) a desire for better management of tension between youths’ needs for structure and wishes for autonomy and (2) lack of RHY input into program governance. This study advances our understanding of RHY, their service needs, and the ways settings meet these needs, as well as remaining gaps. It underscores the vital, life-changing, and even life-saving role these settings play for RHY.

## Introduction

Runaway and homeless youth (RHY) comprise a large and growing population of highly vulnerable adolescents and young adults in the United States (1). RHY emerge from strained and distressed families, characterized, in most cases, by poverty and parental mental health and/or substance use concerns (2–4). Throughout their lives, RHY experience high rates of abuse, neglect, chronic stress, and various other forms of trauma. These adverse experiences generally start in their early lives, while these young people are residing with their families of origin (5, 6). Furthermore, RHY commonly also experience abuse and neglect in the social service systems designed to protect them, and while on the streets (5, 6). These serious negative events and experiences combine with RHY’s lack of involvement in protective systems, such as supportive families, schools, and work environments (5, 7). This confluence of risk factors, coupled with a lack of buffering resources, then results in elevated rates of relational, health, and behavioral problems (5, 7). These difficulties, in turn, place RHY at grave risk for unfavorable long-term outcomes as they enter adulthood. These long-term problems include chronic unemployment, entrenchment in the street economy (e.g., drug dealing, transactional sex/being trafficked), hazardous substance use, incarceration, adult homelessness, unstable relationships, poor health, and even early mortality (8–10). Young people from African American/Black and Latino/Hispanic backgrounds, as well as lesbian/gay/bisexual/transgender/queer youth, are overrepresented in the population of RHY compared with the general population (11). These sociodemographic characteristics inform and complicate their developmental challenges and service needs (12). At the same time, RHY show resilience, that is, the capacity to withstand or recover from significant challenges that threaten their stability, viability, or development (8, 13–15). For example, leaving home is a type of coping response, and is often the best coping response, and surviving out-of-home requires resourcefulness and adaptability (15, 16).

Over the past few decades, a set of specialized settings has evolved to provide services to this population of young people. These include outreach programs, short-term emergency shelters, and long-term programs such as drop-in centers (DICs) and residential facilities called transitional living programs (TLPs). Yet to date, relatively little research has focused on understanding these settings, their effects on youth, and gaps that remain. Some past studies have been conducted on single behavioral interventions for RHY, such as HIV prevention programs (17, 18), and a modest number of other studies have described individual programs or a small number of organizations (17, 19, 20). This study takes a qualitative approach to extend this past research by focusing on RHY clients’ perspectives on their experiences in a diverse set of RHY-specific settings.

**Figure 1:**
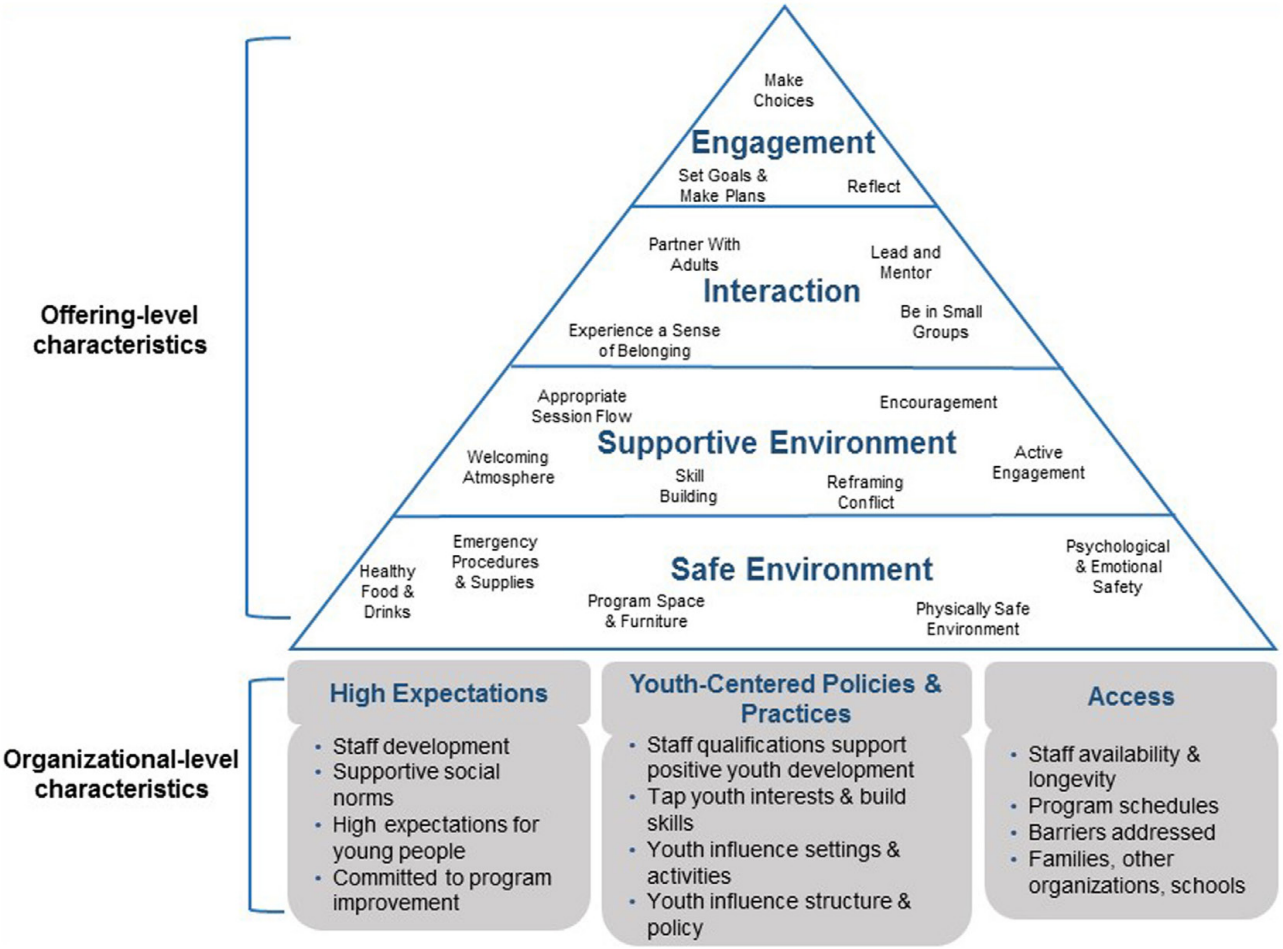
Youth Program Quality Assessment model.

This study was guided by the positive youth development (PYD) approach (21), the accepted basis of programming in most RHY settings (22). PYD is a strengths-based approach emphasizing the importance of youths’ investment in their own goals and the need to promote autonomy and resilience among youth (21). PYD is a youth-centered model in that it prioritizes the needs of youth and the involvement of young people in meaningful ways in the governance of organizations that serve them (21). The Youth Program Quality Assessment (YPQA) model, shown in Figure 1, is grounded in PYD and provides a framework for conceptualizing and assessing non-academic settings designed to promote young people’s positive development (23, 24). The YPQA model examines program quality in two broad categories: offering-level and organizational-level characteristics. Offering-level characteristics refer to the interactions and social processes youth clients experience when engaged in settings (e.g., the extent to which the environment fosters a sense of belonging). Organizational-level characteristics include expectations, policies, practices, and accessibility that support the production of high-quality youth client experiences (e.g., whether the setting has a clear and consistent structure, the extent of youth governance, and input in settings). The two domains correspond to the structure of the typical youth-serving organization: offerings within an organization. Although the YPQA model was not developed specifically for RHY settings, it is appropriate for use in these settings because it is theoretically grounded in the PYD approach.

Given the high prevalence of abuse and maltreatment in the lives of RHY, the settings that serve RHY commonly draw on a trauma-informed care approach (25, 26). Trauma-informed care recognizes the widespread impact of trauma and articulates potential paths for recovery; understands the signs and symptoms of trauma in clients, their families, staff, and others involved with the RHY system; responds by fully integrating knowledge about trauma into policies, procedures, and practices; and seeks to actively resist re-traumatization (27, 28). Thus trauma-informed care and PYD typically serve as philosophical complements to each other in settings for RHY (29). Moreover, because of the adverse effects of trauma on RHY’s abilities to form trusting and healthy relationships, RHY settings commonly attend to the centrality of relationships as a primary mechanism by which engagement, treatment, and positive development come about (30). Attachment theory is one useful means of conceptualizing RHY clients’ relational styles (31). Attachment theory articulates how a strong emotional and physical connection to at least one primary caregiver is critical to personal development (32). Individuals develop an attachment style in response to the characteristics of their early caregivers, with a secure attachment style most strongly associated with positive and stable social relationships throughout the lifespan (32).

We recently took a quantitative approach to describe the characteristics and quality of settings that serve RHY. We focused on a group of randomly selected settings (*N* = 29) across a large but discrete geographical area, New York State (13, 33). We found RHY clients in settings that were rated as higher quality on a quantitative scale showed greater perceived resilience. Furthermore, RHY clients in these high-quality settings were more likely to report being helped with a number of major challenges such as reducing involvement in the street economy (e.g., drug dealing, being trafficked/transactional sex), avoiding or managing substance use, and engaging in school, job training, or work (13). We now extend this past research. Grounded in the PYD approach and the YPQA model, we use qualitative methods to uncover and explore RHY clients’ perspectives on which specific offering- and organizational-level characteristics of these settings play a role in fostering their positive developmental outcomes. Furthermore, we attended to gaps found in settings; namely, the services and setting characteristics RHY clients reported needing but that were lacking. We also explored RHY clients’ reports about how settings can be improved. Given the characteristics of the RHY population, we attended to potential differences in perspectives and outcomes based on racial/ethnic groups and sexual orientation. In keeping with the descriptive and exploratory nature of the study, we do not present formal hypotheses. However, we did anticipate RHY would be more aware of and directly affected by offering-level characteristics than organizational-level characteristics. This is because the latter commonly have a direct effect on setting staff members more so than on RHY clients. Yet, organizational-level characteristics may affect RHY clients indirectly. Study findings will be of interest to RHY service providers, policymakers, and other stakeholders in the RHY community.

## Materials and Methods

### Overview

This qualitative study used a cross-sectional descriptive design and drew on in-depth, semi-structured interviews with RHY clients in diverse RHY-specific organizations across New York State. The definition of RHY varies but tends to include youth and young adults between the ages of 12 and 24 years old (34). This study focused on RHY between the ages of 16 and 21 years of age, who comprise the majority of the RHY population in RHY-specific settings (35). This restricted age range was intended to reduce variability associated with age and developmental level, as the needs and characteristics of the youngest RHY differ from the oldest RHY, while at the same time concentrating on the bulk of the RHY client population. Furthermore, we focused on long-term settings for RHY—DICs, TLPs, and dual/multi-program settings—but not on short-term crisis settings. A setting was defined as an entity providing one or more programs for RHY. The study was conducted in New York State, a region with more than 50 organizations serving RHY, located “upstate,” made up of urban, suburban, and rural areas, and “downstate,” comprise densely populated New York City urban metropolitan area.

### Description of the First Wave Sampling Plan for Settings

In the first phase of this research, conducted in 2015–2016 and described elsewhere (13), we carried out stratified, multistage, random sampling to capture diversity in setting types (namely, TLP only, DIC only, and dual/multi-program settings) and their geographical locations (suburban, rural, and urban areas, the latter both upstate and downstate). From a total of 50 settings, 29 settings that varied in type and geographical location were randomly selected for inclusion in the larger study. In two waves of data collection, we collected qualitative and quantitative data from staff and RHY clients at these settings. After the first wave, we created a multi-perspective quantitative setting quality score (range 0–4) (13). This study drew on in-depth interviews to elicit RHY’s own detailed perspectives on settings collected in a second wave of data collection. Study procedures were approved by Institutional Review Boards at New York University and Solutions IRB.

### Sampling for This Study

From the 29 settings enrolled in the study, we used purposive sampling to select settings that varied in quantitative setting quality scores (to compare data from higher- and lower-ranked settings, a natural contrast) and with regard to location (upstate/downstate) and setting type (DIC, TLP, and dual/multi-program settings). We sampled 11 settings, described in Table 1. In 2016, we conducted in-depth semi-structured interviews with RHY within these settings until saturation was reached on core constructs (36), as determined by a main qualitative researcher and senior staff member who reviewed transcripts. We enrolled 37 RHY clients in this study (mean = 3.36 RHY/setting; range = 2–6 RHY/setting; number depended on size of setting).

**Table 1:** Description of settings (%).

	Drop-in center (*N* = 4)	Transitional living program (*N* = 4)	Dual or multi-program (*N* = 3)	Total (*N* = 11 settings)
**Geographical location**				
Rural	25% (1/4)	25% (1/4)	0%	18% (2/11)
Suburban	0%	25% (1/4)	0%	9% (1/11)
Urban	75% (3/4)	50% (2/4)	100% (3/3)	73% (8/11)

**Region**				
Upstate	50% (2/4)	25% (1/4)	67% (2/3)	45% (5/11)
Downstate	50% (2/4)	75% (3/5)	33% (1/3)	55% (6/11)

**Quantitative ranking**				
Higher ranking	50% (2/4)	75% (3/5)	100% (3/3)	73% (8/11)

### Procedures

Study activities were carried out by a team of experienced master’s and doctoral level researchers from diverse disciplines including anthropology, social work, and psychology with expertise in RHY, qualitative methods, and organizational structures. Research team members made on-site visits lasting 1–2 days each to each of the 11 selected settings. Program administrators informed RHY in advance that study visits would be taking place, and RHY were directly recruited by research study staff in the settings. Each in-depth interview lasted 60–90 min and was audio-recorded and professionally transcribed verbatim.

#### Eligibility

Participants were (1) age 16–21 years; (2) active client of the setting (i.e., had completed an intake at least 1 month ago and attended a program at the setting at least twice in the past month or resided there for at least 2 weeks); (3) approval of an appropriate child advocate if aged 16 or 17 years (i.e., an adult staff member with knowledge of the young person because RHY clients aged 16 and 17 years, as minors, cannot provide signed consent for research activities); and (4) not currently in foster care (because youth in foster care cannot participate in research without the consent of the department of social services). Note that youth enrolled in foster care are not commonly found in settings for RHY.

#### Consent/Assent

Runaway and homeless youth aged 18 years or older gave signed informed consent for participation in the in-depth interviews. We obtained a waiver of parental consent from the Institutional Review Board for RHY ages 16 or 17 years. These young people provided signed informed assent, and we also elicited the approval of a child advocate in the setting in the form of a signature on the assent form, before the youth’s participation.

#### Compensation

Runaway and homeless youth clients were compensated $30 for their participation in the in-depth interviews.

### Measure (Semi-Structured Interview Guide)

We used a semi-structured interview guide grounded in the YPQA model. The interview guide explored RHY clients’ backgrounds and paths to homelessness (for context) and perspectives on the offering- and organizational-level characteristics of the setting. With respect to background factors and the path to homelessness, the semi-structured interview guide included questions on the participant’s concept of “homelessness,” first experiences out-of-home, relationships with family, and experiences with and perspectives on RHY service settings, beginning with the RHY client’s first experience with such settings. Regarding offering-level characteristics, the interview guide focused on the setting where the RHY client was recruited for this study and probed for his/her perspectives on strengths and weaknesses in the setting, striving to go beyond the socially desirable response and also to elicit emergent and unexpected themes. Grounded in the YPQA model, the guide included questions on the extent to which the setting provided an emotionally and physically safe environment; the extent to which RHY clients felt supported in the setting, and how that support was provided (including relationships with staff and other RHY clients); aspects of interactions with and relationships between RHY clients and staff; and the extent to RHY clients’ involvement in their own goals and future plans. Thus, the YPQA model is positioned to document the use of a trauma-informed care in settings, as the model attends to relational/attachment aspects of settings. Some organizational-level characteristics may not be apparent to clients in a setting, but some indices on the YPQA are expected to directly affect clients. Thus, the interview guide posed questions about organizational-level characteristics such as staff members’ expectations for RHY clients, whether RHY clients have influence over programmatic activities and/or governance of the setting, and factors pertaining to access. Specific examples were elicited from participants where appropriate. Sociodemographic information (age, sex at birth, race/ethnicity, sexual orientation, and gender identity) was collected for each participant.

### Data Analysis

We analyzed the qualitative data using an approach that was both theory-driven and inductive using the Dedoose platform (Dedoose Version 7.0.23, 2016), taking a systematic content analysis approach (37). The analysis process began with the generation and application of a robust set of reliable and valid codes. First, the research team created a “start code list” based on the research questions and domains of the YPQA model and PYD (38). These codes were comprised of labels or tags (containing one to several words) assigned to sections of text (words, sentences, paragraphs) that were accurately described by that code. First, a main data analyst read through four interviews and applied the start list codes to segments of text. This analyst created new codes based on emergent themes relevant to the main research questions or that were repeated in the transcript or across transcripts. Then, a second analyst independently coded a selection of excerpts already coded by the first analyst. The two analysts worked closely to discuss codes and establish inter-analyst reliability. Discrepancies in coding were resolved by consensus. Through this grounded and inductive approach, additional codes emerged, and the codebook was further elaborated and refined (39). Analysts attended to potential differences in perspectives and outcomes based on racial/ethnic group and sexual orientation (heterosexual orientation vs. lesbian, gay, bisexual, queer, or other non-heterosexual orientation). Once consensus between the two analysts was reached on a consolidated list of codes and their definitions, both analysts revisited the interview transcripts they had already coded and incorporated the final list of codes. The first analyst then coded the remaining transcripts, the second analyst also coded approximately 25% of them, and discrepancies were resolved by consensus.

Next, emphasis shifted from coding to identifying larger themes. The full analytic team comprised of the two data analysts and senior research staff formed an “interpretive community” (40), which engaged in an iterative analytic process. The analytic included regular meetings to discuss the most frequent and resonant codes, relationships among codes, and their explicit and underlying, latent meanings, which were combined to form unifying themes. For example, resilience (a construct of interest as noted above) was not an explicit code but instead formed a latent theme. Codes and themes were deemed primary when they were introduced or discussed by numerous participants within a setting, or when they emerged from participants across multiple settings.

#### Methodological Rigor of Data Collection and Analysis

We attended to the methodological rigor of the data collection process through periodic review of transcripts and process memos written at the time of each setting visit to attend to fidelity to the interview guides and quality of data and thereby ensure consistency across interviewers. Furthermore, the senior team members conducted regular debriefing with the research field team, and a review of transcripts was conducted by an expert in RHY settings (41). Methodological rigor of the analysis was also maintained through an audit trail of process and analytic memos and periodic debriefing with the larger research team, which included experts in settings and RHY (42).

## Results

### Description of the RHY Sample

As shown in Table 2, RHY were 20 years old, on average (SD = 2 years). With respect to race/ethnicity, 42% were African American/Black, 31% were Latino/Hispanic, and 28% were White/other. Half (54%) were female sex at birth. Among the participants born male (46%), five had a male-to-female transgender gender identity. Almost half of participants (49%) identified as lesbian, gay, bisexual, queer, or otherwise non-heterosexual.

**Table 2:** Runaway and homeless youth client sociodemographic and background factors [Mean (SD) or %], *N* = 37.

	M (SD) or %	*N*
Age in years (M, SD)	20 (2)	–
Race/ethnicity		
African American/Black, not Hispanic	42	15/36
Latino/Hispanic	31	11/36
White/other, not Hispanic	28	10/36
Biological sex at birth		
Female sex	54	20/37
Male sex	46	17/37
Gender identity		
Transgender gender identity—female-to-male	0	0
Transgender gender identity—male-to-female	14	5/37
Lesbian, gay, bisexual, queer, or other non-heterosexual sexual orientation	49	18/37

### Overview of Qualitative Results

As detailed in the analysis below, RHY clients identified a number of meaningful ways in which RHY-specific settings cultivated a sense of optimism, resilience, and feelings of wellbeing, as well as enhancements in behavioral functioning. Moreover, these young people provided their perspectives on some ways in which settings, even higher-quality settings, could improve. Overall, we found the specialized nature of RHY-specific settings was critical to the effectiveness of their work with RHY. Particularly, from the perspectives of RHY, a population-specific approach tailored to their specific needs was apparent in these settings, from the physical environment, through a particular skill set of staff, and the theoretical approaches informing services and treatments. This tailored approach reflected the fact these highly vulnerable youth typically presented to settings with a great sense of distrust of both professional adults and social services. This distrust complicated RHY clients’ processes of engaging in settings, as well as their ongoing experiences with settings. Indeed, we found RHY had experiences with various types of settings over their lifetimes, some RHY-specific and some not, and reported the greatest benefits from RHY-specific programs, as described below.

With respect to the primary specific positive effects of RHY-specific settings on these young people, analyses revealed four main themes. (1) The process of first engaging with an RHY setting was experienced with trepidation and was fraught with emotion and meaning, and the experience of safety within RHY settings was critical to facilitating young peoples’ transition out of acute crisis and into service provision. (2) Instrumental support; that is, tangible help and services, was seen as vital and was most effective when received within the context of emotional support. (3) RHY clients showed a high level of skill related to survival outside of the home, on the street, and away from the guidance of their family of origin. Yet, competencies to thrive in other settings were less evident. Therefore, RHY settings were critical for RHY to build resilience, optimism, and confidence regarding the possibility of future successful engagement with the larger society. (4) Follow-through and aftercare were seen as vital as RHY clients transitioned out of service provision.

Regarding RHY’s perspectives on gaps in services and needs for program improvement, the main themes in this analysis were (1) the need for settings to better address the tension between RHY’s perceived need for structure and their desire for autonomy and (2) a lack of understanding of and input into program governance among RHY in many settings. Names used below are pseudonyms, and some identifying details have been changed to protect the confidentiality of both RHY clients and the settings. For context, in the sections below, we provide a number of sociodemographic characteristics describing participants who are quoted, including age, sex at birth or gender identity if transgender, and race/ethnicity. (RHY were cis-gender unless otherwise noted.)

### Part I: Beneficial Effects of RHY-Specific Settings on RHY Clients

#### RHY’s Complicated Pathways to Settings

Runaway and homeless youth typically described their past circumstances, and in some cases, their current situations, whether they thought of themselves as “homeless” or not, as characterized by chaotic and unstable environments that disallowed the physical or emotional space needed to adequately reflect on their current situation, and consequently, impeded their abilities to imagine a different, and perhaps more desirable, developmental trajectory. (In the following sections we refer to RHY as “homeless,” meaning they resided without parental/guardian supervision in temporary locations, institutional placements, or places not intended for habitation, and we acknowledge that some RHY did not use this term to describe themselves.) In particular, many RHY described that before engaging with RHY-specific settings, they existed crisis-to-crisis, attending only to survival and basic needs. For most, this overall sense of living in chaos and instability stemmed from the experiences of multiple and overlapping traumas perpetrated by families, individuals they encountered in the street, and police and other authorities; this sense of chaos was often complicated by poverty and the need to cope with these experiences to survive. Specifically, these traumas were described as common in both their earlier and more recent lives. Traumas reported ranged from experiences of physical, emotional, and sexual abuse; physical and emotional neglect; non-acceptance of their sexual orientations and/or transgender gender identities; extreme scarcity of resources (e.g., lack of food, clothing, and supplies to maintain good hygiene); mental health and substance use problems; legal and immigration issues; and police targeting and/or harassment.

The process of becoming homeless and engaging with RHY-specific settings was typically complicated and fraught for RHY. A substantial proportion of RHY gradually but perceptibly transitioned from residing with their families to either street homelessness or unstable housing. For example, RHY typically left and then returned home a number of times or were forced to leave home a number of times, before finally realizing they could not or were not permitted to return. Yet, we found these young people typically did not connect emotionally to that reality. Nor did they fully understand they were, in fact, engaged in a process of becoming disengaged from the care of their families, leading to the state of being homeless. Most RHY described the initial realization they were actually out-of-home or homeless as “a complete shock.”

Runaway and homeless youth’s initial reactions to homelessness were particularly important as youth first began to experience various aspects of RHY-specific settings. A lack of emotional connection to the process of becoming homeless often contributed to a situation wherein the young person’s experiences, needs, and treatment goals were both difficult for them to articulate to RHY settings and highly variable. This, in turn, complicated the engagement and treatment efforts of RHY settings and created a need for programmatic flexibility to assist RHY to set goals and understand expectations, whether on a more micro, immediate interpersonal/behavioral level or on a more macro level, dealing directly with a youth’s long-term life trajectory. Indeed, a deep understanding of RHY’s backgrounds and the effects traumatic life experiences was critical for staff within RHY settings to provide a sense of physical and emotional safety and to build relationships with individual youth.

When reflecting on their initial engagement with RHY-specific programs, RHY typically reported feeling trepidation, resistance, and fear, and anticipated being treated poorly or even being in physical danger upon entering these settings. Many RHY had preconceived, negative views of social service settings in general, and often conflated any type of program dealing with homelessness, either for youth or adults, with stereotypical warehousing of people into dangerous situations. Indeed, many older RHY already had some experience with adult treatment settings and family shelters in their very early lives. This, and the fact that many programs were seen as a poor substitute for a loving and supportive family, made making the transition into even RHY-specific settings particularly challenging for RHY. Olivia was a 21-year-old African American transgender woman. She described,

I honestly (at first) felt more safe in the street than I felt in one of those shelters … I did an intake asking for all my problems, and I’m like no, no, no you’re not going to get me anywhere. Just like, the atmosphere. It was brand new to me. I’d never been to that. So, coming to that environment was like … I’d rather be on the streets because I know the streets. I don’t know the shelters.

Thus, particularly early in their transition into homelessness, RHY clients commonly reported strong, often ambivalent, or even negative, feelings about social service settings, which served as an impediment to their engagement with RHY-specific settings, even those that could provide vital housing services. As Kayla, a 21-year-old White female notes,

When I thought (about) a shelter, I thought of like a big room with bunk beds that people sleep in at night. That’s what I thought. And I didn’t want to do that. But when my guidance counselor had told me about [the RHY-specific TLP], and told me it was a house setting, I went for the intake, which is like an interview. And they accepted me. And I came. And from there I stayed.

We found that RHY clients did not, in fact, experience RHY-specific settings as at all comparable to adult shelters or service settings. Instead, RHY generally experienced RHY-specific settings, particularly DICs and TLPs, as safe, and in many cases, “homey.” This was vital because RHY clients, as would be expected, felt the loss of their families and homes acutely. Indeed, before their initial engagement, RHY-specific programs were often associated with the emotional chaos RHY were experiencing as they transitioned to homelessness, the loss of their families, and then the realization they were officially out-of-home, resulting in initial hesitancy to engage with programs. In this context, RHY clients prioritized the need for a physically and emotionally safe environment in which they could begin to assess their current situations and to better manage the transition from homelessness to engagement with other types of services and/or into transitional housing. For example, Olivia, mentioned earlier, compared her initial apprehension with her actual experience with an urban TLP:

A lot of people, they think of a shelter and they probably immediately think of this really dark, dreary kind of place. And it’s true for most places, but [this RHY] shelter is set up like a home. So it’s really a fun, welcoming environment that really helps you, and supports you, and listens to you. So it’s really awesome.

Within this context youth noted they were able to work with staff and even other youth to take the time to gradually identify and address their individual issues, and that this time and space were instrumental in this transition from the streets to an RHY setting. Indeed, we found young people repeatedly distinguished between RHY-specific programs and other types of shelters and group homes, such as foster care settings, consistently reporting that RHY-specific programs were better able to understand them and meet their needs. Thus, we found RHY’s experiences with and knowledge of various both youth and adult shelters and service settings provided a useful contrast to their perspectives on RHY-specific settings, and thereby highlighted the benefits of RHY-specific settings such as crisis shelters, DICs, and TLPs.

Runaway and homeless youth clients almost universally viewed RHY-specific programs as unique in their abilities to provide a safe and at least relatively stable environment, in stark contrast to RHY clients’ experiences with abusive families, street homelessness, and to some degree with adult shelters. For example, Ronald was a 20-year-old African American man with past experiences with street homelessness in a large urban environment, adult and youth crisis shelters, and with RHY-specific programs, which he compared and contrasted:

Yeah, you know, like [RHY programs] help you become more independent. It’s … more like a process. It takes time. And it’s actually, it’s time that you can actually deal with and you can actually handle … Once you get kicked out of your house, … you’ve got to develop that mindset, to survive. At least here, at [this RHY program] it’s like you have time to actually think about stuff, whereas on the street or at [an RHY crisis shelter], it’s more like you’re in survival mode. So, … it’s good to actually have some space to actually breathe. Sometimes I’m just here, just thinking about life in general. You know, like how the world works and why people think the way they think … So, it’s like being in this [RHY program] environment, it’s possible to think about those things … [Here] it’s more of a peace of a mind. You’re just quiet.

Similarly, Jessica, an 18-year-old pregnant Latina woman living in a TLP that serves pregnant RHY and youth mothers, contrasted her experiences in youth shelters with the “family-like” environment of long-term RHY programs that were characterized by a sense of safety and warmth.

Yes, it’s actually really nice there. At first, I was really hesitant to go ’cause I been in the shelter since June of last year, and I was kind of getting sick of shelters. And once I got here—it’s better than a shelter. It’s more homey … They’re more considerate and understanding. I thought it was going to be like, I’m gonna have to watch my back 24/7, watch my things. But here I can leave something out and it won’t disappear. I can talk to any of the (staff) and they won’t get an attitude or get mad. It’s just a friendly and welcoming environment, and I didn’t think it would be … It’s better than a (youth) shelter. It’s more homey … Not only is their food way better, but they’re more welcoming. More of a homey environment.

Thus, RHY clients stressed that the tailored environments and modes of engaging in RHY-specific settings were critical to their willingness and ability to transition from crisis and chaos into receiving services. However, youth crisis shelters were challenging environments for many because of the large numbers of RHY in a state of crisis and chaos, the short-term nature of placements, and the lack of stability to allow newer RHY to find peer role models and guides. Yet, crisis shelters were the vital bridge to the next step for RHY, whether that was to return home or enter a long-term facility such as a TLP.

#### Integrated Instrumental and Emotional Support

Once youth had made the transition into receiving RHY-specific services in DICs and TLPs, they reported benefiting greatly not only from the safety and security there, but also from the various resources and programs these settings provided. In these RHY-specific settings, RHY clients received basic necessities such as food, clothing, and housing, and staff also helped them prepare to apply for jobs, continuing education, housing, and other programs for which they might be qualified. For most RHY clients, simply having staff to assist them in navigating the otherwise daunting world of non-RHY social services was vital. For example, RHY clients typically needed help obtaining state-issued identification required for applications for school, employment, and public assistance benefits, all of which are essential to transitioning out of homelessness and becoming more self-sufficient. While obtaining identification is a relatively straightforward task for adults and adolescents generally, RHY clients typically had no access to needed documents such as birth certificates, and they often lacked familiarity and comfort with large bureaucracies designed to provide services to adults. Indeed, RHY clients commonly described themselves as well socialized into the norms of the street, but as less able to navigate more conventional settings.

Notably, many RHY clients reported that emotional support was a critical complement to instrumental support. This emotional support helped them overcome bureaucratic obstacles and to prepare for potential rejection. RHY clients commonly referred to RHY-specific programs as operating as a type of surrogate family, and youth repeatedly cited benefiting from such instrumental support as transportation costs, referrals to other programs such as vocational training and mental health services, and programs that catered to RHY clients’ individual interests (e.g., art and music programs), always within the context of emotional support and one-on-one guidance. As Maya, a 20-year-old Latina transgender woman with multiple years of experience in both DICs and TLPs noted,

I came into this program with basically nothing. No clothes, no food, no money. And I didn’t basically have anything to fall back on. And they made sure that by the time I got to where I am now that I have everything that I need to make sure that I’m independent, an independent youth. And being at a young age like 14, 15, 16 years old, not a lot of kids survive. And [they] would usually have nothing if they went through something that I’ve been through. And I just can say it’s been a blessing to have them support me through it all. They’ve helped me get back in school to get my GED. They’ve helped me find jobs. Helped me become more independent with myself, and be comfortable in my community. They’ve helped me find apartments. I don’t think I’d have made it this far on my own if I never knew about them.

Throughout interviews with RHY clients in both DIC and TLP settings, RHY consistently highlighted the ability of RHY-specific programs to provide the thoroughly integrated instrumental and emotional support that distinguished these programs from non-RHY programs, and which they ultimately said provided the greatest benefit to them through their transition out of homelessness.

#### Resilience, Optimism, and Confidence for Successful Adulthood

Fostering resilience, confidence, and optimism among RHY clients, within the context of their past and ongoing traumatic experiences, was a key function of RHY-specific settings. As many young people noted, months or even years of nearly uninterrupted trauma and relative social isolation left them suspicious and distrustful of others. For example, RHY clients often described themselves as feeling unbearably anxious, confused, or “shy” in unfamiliar settings. For many RHY clients, and especially those who reported a lengthy history of abuse, developing confidence in their abilities to succeed outside of the streets and RHY-specific settings often translated into the capacity to simply have the courage to ask for assistance when necessary. For most, this was made possible by the ability to have prolonged interactions with other RHY clients and with staff in a safe and supportive environment wherein self-expression was valued, and de-escalation of conflict and positive communication were actively taught. Jessica, mentioned earlier, described her experience as follows:

Before I became homeless, I was never the type of person to ask for anything. I was so shy I would never ask. I would never ask, I barely listened, and I was just the type of person that liked to do things on my own. But when it comes down to it, my mom would do whatever I didn’t know how to do. So, I decided to take a different route and go out of my comfort zone and do what I had to do to get myself up there in the world … [In our groups in the TLP] I feel like I could talk about anything around them, like, we’ve got so much in common. But I’ve always thought [groups] were weird until I moved into the [RHY setting] and now I’m just ready to ask questions. Questions popping up in my mind, I ask, and I tell them experiences I’ve had or things I’ve heard, and I’m looking for answers and I’m asking questions.

Similarly, Ronald the 20-year-old man described earlier, stated: “*Because in life, you know, you’re always gonna have questions. So you have to learn how to ask questions, when to ask questions. Even if it’s something that you think is the most obvious thing—ask the question*.”

For others in DICs in particular, simply being able to interact with similarly experienced peers in a relatively stable environment was enough to allow social skills and confidence to begin to develop. Tabatha, a 21-year-old White female who frequented a rural DIC, noted,

It’s fun and kind of brought me out of my comfort zone. I was always nervous meeting people. I was always too shy. I really like staying in my room, reading stories, writing, and stuff like that. But [my boyfriend] brought me here and kinda made me come out more and meet new people and things like that. Well I started to talk more and stuff like that. I started feeling not so shy and stuff like that. I started talking, doing more active and stuff like that … Um, maybe like I wanna walk out more and go places and stuff like that … Probably meeting new people and Like seeing how some people are nice and stuff like that.

Again, citing the negative psychological effects of chronic homelessness, others reported the emotional space provided by TLPs led even more directly to developing self-confidence and resilience. Angeleae was a 19-year-old African American woman who described,

So one thing I learned right off the back is never tell myself no. Before I even moved to New York State, I had a long history of homelessness with my mother as a young child … Another thing I did to assess my life was [to stop] speaking negatively about myself to myself. I didn’t so much focus on the outside at first, but I really looked at me on the inside and how I really felt about myself. And that’s where I started to change my life. In [the TLP].

#### Follow-Through/Aftercare

Finally, RHY clients who were considered to be “graduated” from a TLP expressed a strong sense of appreciation of knowing that they had the ability to reach out to RHY staff even after having “aged out” or “timed out.” Youth and staff alike repeatedly stressed the importance of having a clear understanding that the instrumental and emotional support that characterized the RHY client’s time with the program would never completely terminate. As Madison, a 20-year-old transgender woman at a semi-urban TLP described, “*I’m at the last part of it all. So, they’re still gonna help me, and they’re not gonna stop until I reach my goals. And I like that about the (RHY setting)*.” Similarly, Aisha, a 21-year-old African American woman living in a TLP noted,

Once you leave one of their programs, they’ll still check in with you, even if it’s through text or they’ll give you a call or e-mail you. They’re trying to reach out any way they can and ask you, how’s it going? What’s going on? Are you guys getting along? Just different things along that nature. Do you need any money for food or anything like that. So, they’re still there for you in a capacity they can be. And I do believe they have a program, like within the [RHY setting] and that’s like their whole goal. I think it’s called Aftercare or something like that.

Given the paucity of long-term supports in RHY clients’ lives, the support of understanding and caring adults, even after they age out of services, was comforting for RHY clients and increased their sense of confidence that they could survive in their post-RHY-service world. Just as families provide support to their emerging adult offspring, RHY settings maintained connections with former clients as they moved to the next developmental level.

### Part II: Gaps in Services

#### Tension Between Structure and Autonomy

In the following section we describe RHY clients’ perspectives on ways settings could improve. One of the most influential factors in RHY settings was the successful navigation of the complex tension between young peoples’ needs for structure and their desires for autonomy. Yet, this balance was not easy to achieve, and the mechanisms through which this balance came about were not always apparent in youths’ statements. While many RHY clients were eventually aware that this tension between support and autonomy was perhaps inevitable in these settings, many youth, and particularly those who were still adjusting to life in an RHY-specific setting, indicated some of the ways staff could do more to help RHY clients understand and benefit from a structured environment. For example, youth commonly recalled that as they began to develop confidence to question the conditions within which they were living, they gradually began to feel a need for increased autonomy, which, in turn, provoked a sense of unease related to their previously identified need for structure and security.

This was particularly evident in statements made by youth in TLPs. RHY clients in TLPs frequently moved fluidly back and forth between statements indicating extreme gratitude for the emotional and instrumental support of the staff on the one hand, and slight contempt for what they often described as overbearing and unnecessarily strict guidelines on the other. This tension is evident in how Evelyn, a 16-year-old White young mother at an urban TLP, described being supervised by TLP staff.

They run it like they’re your parents and you’re supposed to tell them where you’re going, how you’re going, when you’ll be back. Like those are parenting things. That’s what they do. [But] I shouldn’t have to come back and have to tell you guys like, oh, I was here with this, this, and this. That’s what my parents are for.

This tension was exacerbated in many cases by young people’s ambivalent feelings about being supervised within an RHY-specific setting by someone other than their parents, and, in many cases, caused ambivalent feelings about wanting to reunite with their parents. As Nathan, a 19-year-old White man in a semi-urban TLP pointed out,

You know [some RHY] wish they had [a room in a TLP], you know, what we have, because not everybody has it. But, I tell the people that it’s good, but then it’s not, because people want to be able to be on their own and do things on their own, instead of having to be in here. I like it, but then I don’t, because I want to be able to be on my own, and I have to be in a group program, where I’m still being watched because I kind of feel like I’m in my parent’s house. But I mean, other than that, the program is perfect. They help you with your GED classes, they try to make goals for you. Because if I don’t get on top of the goals, they’ll try to push you to gain that enthusiasm to go do those things even if you don’t want to.

Many RHY clients also expressed this tension between the need for autonomy and support in terms of a frustration with other TLP residents who they perceived as less determined to meet their stated goals, and therefore as less deserving of equal services, and sometimes even less deserving of autonomy. One common theme was the tendency for youth to locate themselves and one another along a continuum of maturity that seemed dependent on how they viewed themselves and others engaging with the program in an earnest and purposeful manner. One of the ways that youth attempted to comparatively locate themselves along this continuum was through articulating a clear desire for self-sufficiency, in contrast to behavior that was referred to as “freeloading” or “taking advantage” of what the program had to offer. Indeed, for some, being perceived as genuinely desiring of self-sufficiency, which was equated with “normality” by many, might even be seen as a way of beginning to truly distance themselves from the stigma of “homelessness;” however, this term or situation might be conceptualized. As Nathan, described earlier, noted,

They see that [living in the TLP is] good because you get to [have a home], and use free electricity and all that other stuff. But that’s not the way that I look at it as. I look at it as, it’s an alright thing. But then it’s not. [Some] people want to be out on their own, and there’s people that don’t. I’m one of those people that want to be on my own. I mean, it’s not a good thing to me that I use the [electricity] here. And that, you know, I put nothing into [the TLP] moneywise because, of course, [electricity] cost money. After a while of you being in here, you feel like you’re not doing nothing for [the TLP]. I mean besides doing what they ask you do to. That’s just not enough … I want to feel like a normal person that is out on their own. I don’t feel like I’m doing enough, or giving enough.

Nonetheless, given the gratitude that most youth showed for even the most basic services and support, many noted they were willing to capitulate to what they considered to be inconvenient or even unreasonable demands rather than face the street or a shelter again. As Jessica, the young pregnant woman mentioned earlier, noted,

The only thing that I didn’t like was that we had to be back at certain times for meetings, like, at five o’clock or four o’clock, and then we can leave again. But curfew times were, like, 10 and 11. But once I ended up going there, I decided to give it a chance. I had about a month left till I was having my baby, and the [RHY-specific setting] was pretty much my only option left.

#### RHY Client Program Governance

Runaway and homeless youth client input into governance is a core tenet of the YPQA model that guided this study and a strongly held value in many RHY settings, consistent with the PYD approach. Yet, we found, aside from a small number of examples, youth input was limited to mundane household rules and decisions, making chore assignments, talking out interpersonal problems among residents, and settling disputes over issues such as internet access and usage. Moreover, and closely related to the internal and interpersonal debating over exactly what balance of structure and autonomy is appropriate for youth transitioning out of homelessness, one of the most notable areas for program improvement regarding youth’s psychosocial development had to do with the youth’s own understanding of the ways in which programs operated on an institutional level. In fact, a lack of understanding of the reasoning behind the perceived “strictness” of basic rules and regulations at times even lead to misunderstandings between staff and RHY. For instance, despite having an overall appreciation for the TLP in which she resided, Evelyn, the 16-year-old young mother described earlier, expressed such a degree of frustration over what she regarded as unnecessarily prohibitive requirements that she began to compare the TLP to non-RHY-specific homeless shelters or group homes:

If you’re not working or at school or we’re not in groups, we can go out and do what we want, but there’s only like certain times. Like say I was out for probably an hour and a half, two hours. And then they told me I had come back and then I have to do chores and stuff like that. I used to live in group homes. I didn’t realize it was so much of a group home [here] in that we had to do things along that nature. In a homeless shelter you don’t do things like that. [In shelters] you come and go as you please. And [here] you’re so tired.

Indeed, a lack of RHY’s awareness and understanding of the reasoning behind many DIC and TLP rules and regulations was a recurring theme that pointed to a lack of communication between youth and staff regarding the institutional decision-making processes.

Most strikingly, throughout the majority of interviews, even when explicitly asked about the degree to which they were able to genuinely contribute to decision-making on this level, youth were frequently confused or needed time to respond, and the vast majority seems to have never considered themselves in this role. Indeed, most RHY clients did not seem to understand that their input could be something valuable to the setting. As one youth tellingly put it, “*we just comment*.” However, it is unclear from many of the youth’s comments how this ambivalence relates simply to a lack of effort on the part of program staff to engage with youth at this level, or whether the lack of ownership over programs is something in which youth simply are not interested, or both.

## Discussion

The study of the ways RHY-specific settings influence the lives and developmental trajectories of RHY and the mechanisms by which settings achieve those objectives, have received little attention in the research literature to date, particularly from the perspectives of RHY clients themselves. This study, guided by the YPQA and the PYD model, takes a descriptive qualitative approach to address these important research questions and thereby extends past research on RHY and RHY-specific settings. In addition to uncovering settings’ positive effects on RHY, as we discuss below, we further identify some specific organizational characteristics and services that RHY need, but that are lacking in settings. We also uncovered youths’ perspectives on ways in which RHY-specific settings, even those demonstrating higher quality, can improve. Importantly, we focus on youth in a diverse set of RHY-specific settings, which varied with respect to geographical location, type, services provided, and quality, which, we found during analyses, fostered the validity of study findings and their utility.

### RHY-Specific Settings Are Tailored to the Population, Promoting Effectiveness

The challenges RHY face throughout their lives, and resultant complications these create for locating, engaging, and serving them, are well documented in the research literature. Indeed, some of the most concerning effects of maltreatment and trauma experienced by RHY are distrust and fear of professional adults and service settings, which causes RHY to avoid presenting to or engaging with settings (43, 44). We found that RHY experience RHY-specific settings as oriented toward addressing these serious relational issues on a number of levels.

First, we found settings are positioned to build positive, developmentally appropriate, and professional relationships with RHY. This emphasis on the centrality of relationships is consistent with attachment theory (32). In a past study of vulnerable young men that included RHY, we found that a non-secure attachment style was associated with young people remaining outside of the protective systems of services, family, school, and work, and also associated with risky contexts such as the street economy where they are less likely to encounter prosocial peers and adults (31), a finding echoed in other studies of attachment among RHY (45). Importantly, however, positive relationships can develop among those with non-secure attachment, and one’s attachment style can even evolve (46). Study findings suggest RHY-specific settings are actively oriented toward addressing these types of interpersonal challenges to thereby foster engagement, even when RHY experience difficulties with trust and relationships. Second, study findings highlight the primacy of trauma in the lives of RHY, and underscore the utility of a trauma-informed care approach. Trauma-informed care is a strengths-based approach that includes awareness of trauma among RHY and also secondary traumatization for staff, an emphasis on safety, and opportunities to regain control (26). We found RHY’s perspectives on settings are consistent with a trauma-informed care approach being implemented in settings. Finally, many elements of PYD, a philosophical foundation for most RHY settings, resonate strongly with RHY, including its emphasis on strengths, similar to trauma-informed care, and youth autonomy and participation in setting their own goals. Yet, balancing support and autonomy can be a delicate balance in RHY settings, as we found in this study, and as we discuss in more detail below.

In sum, this study findings suggest the utility of an integrated service approach and philosophy focused on relationships, the sequelae of trauma, engaging RHY in their own goals, and identifying and fostering strengths to address the needs of this complex population of young people. RHY experience these RHY-specific settings as purposively designed to address the barriers they face to service engagement and psychosocial change. Indeed, RHY experience these settings as uniquely positioned to engage and serve them, and as a result, as more useful to them overall than general adult or non-RHY youth settings. This specialized, tailored approach encourages RHY to engage with services they otherwise would very likely never come into contact with or would avoid and allows for the receipt of integrated instrumental, emotional, instructional, and quasi-familial support critical for transitioning out of homeless and into a safer, healthier, and more satisfying life trajectory. This study identified the ways settings assist RHY and also the mechanisms by which settings do so; for example, providing instrumental support integrated with emotional support and helping RHY build skills to interact with the systems they will need to master to function independently when they age out or time out of their present placements.

### Advancing Research on the YPQA

As noted earlier, the YPQA was developed for youth after-school settings, and this study provides some support for the utility of the YPQA model for the study and evaluation of RHY-specific settings. With respect to offering-level characteristics of these settings, RHY highlighted the importance of a sense of physical and psychological/emotional safety they experience in RHY settings, particularly in the long-term settings. RHY view setting environments as supportive, and in particular note the positive effects of encouragement and skill building. They value settings as being home-like, and staff for standing in for family. Finally, engagement is evident in RHY clients’ involvement in their own goals and plans. In past research, we studied these offering-level characteristics using quantitative coded observations of programs within RHY-specific settings (13). The YPQA was developed for after-school programs, but not specifically settings that serve RHY. Thus, some domains of the YPQA model are not typically evident in RHY settings because the structure of activities varies from those found in after-school settings. Furthermore, some aspects of important offering-level characteristics in RHY settings were not captured by the YPQA. To address these limitations of the YPQA, this study highlights the utility of eliciting RHY’s perspectives on offering-level characteristics, in addition to those captured by the YPQA. Indeed, in this study RHY provide insights into the aspects of settings that have the greatest influence on them and the mechanisms by which settings make a difference in their lives.

Organizational-level characteristics are generally assessed from the perspectives of staff, and may not always be evident to RHY (e.g., whether the setting has high expectations for staff). Yet, in this study, RHY did have some insights into organizational-level characteristics and highlighted both strengths and gaps. Findings reflect the salience and importance of youth-centered policies and practices in settings, an important component of PYD. While RHY note this philosophy in practice, for example, with respect to flexible goal setting, they also experience tension between their needs for autonomy and structure; for example, with respect to rules and regulations in some settings. In fact, balancing adolescents’ and young adults’ needs for autonomy with support and guidance is a challenge for all youth-serving settings, as well as in families (47, 48). This balance may be particularly challenging in RHY settings, however, because RHY have generally become accustomed to living independently, unaccustomed to guidance from caregivers, and wary of professional adults.

### Gaps in RHY-Specific Settings

Runaway and homeless youth highlighted a general lack of youth involvement in the governance of settings; that is, they do not typically have input into the settings’ policies and practices. Yet, youth involvement is a key feature of the PYD approach and may be one critical aspect of fostering a sense of autonomy and engagement among youth and reducing frustration and tension between staff and RHY clients in settings. Furthermore, youth involvement/youth governance may be a potential solution to addressing another gap in settings identified by RHY clients; namely, the challenge of balancing RHY clients’ needs for autonomy with rules and expectations in settings. Thus, RHY clients and staff could work together to create rules and policies, which may, in turn, be more acceptable to RHY clients than those they experience as imposed upon them. Moreover, RHY express trepidation about aging out or timing out of services before they are ready for independent living, suggesting that program constraints may not always align with youths’ needs.

### Limitations

The study focused on long-term RHY settings in a single geographic area in the United States, which may limit its generalizability. Furthermore, the non-random sampling method of RHY within settings was a potential limitation, as it may have introduced social desirability and other biases; for example, if only RHY with uniformly positive views of the setting were recruited. The triangulation of findings of youth within and across settings was used as a strategy to reduce such biases. Furthermore, as we described earlier, African American/Black and Latino/Hispanic young people and/or those with transgender gender identities and/or lesbian, gay, bisexual, and other non-heterosexual sexual orientations are overrepresented among the population RHY compared with the general population (12, 29), and most RHY participants in this study were from these social categories. Yet, this analysis did not yield themes related to race/ethnicity, sexual orientation, or gender identity, although participants were typically queried about the influence of these social categories on their experiences and wellbeing. Further analyses should use innovative methods to elicit findings related to race, ethnicity, and other social categories. Furthermore, this lack of such findings suggests the utility of an intersectional approach; that is, the interconnected and non-additive nature of social categorizations such as race, class, and gender as they apply to a given individual or group, where social categories cannot be understood separate from each other (49). There is growing awareness of the value of an intersectional approach to advance public health research (50), but intersectionality is only beginning to be applied to the study of RHY (51). Furthermore, better tools may be needed to adequately capture RHY setting quality; the YPQA could be modified, other tools can be identified and refined (52), or new tools can be developed.

## Conclusion

This study advances our understanding of the population of RHY, their service needs, the ways in which settings meet these needs, as well as gaps that remain. As such, it underscores the vital, life-changing, and even life-saving role these RHY-specific settings play in the development and wellbeing of this complex population of multiply challenged young people.

## Ethics Statement

The protocol was approved by the IRB at NYU. All subjects gave written informed consent in accordance with the Declaration of Helsinki.

## Author Contributions

MG conceived of the overall study concept and design and writing the manuscript. RF planned the qualitative data collection effort, collected the data, led the effort to analyze and interpret data, and helped write the manuscript. AK collected data and played a leadership role in analyzing and interpreting data, as well as reviewing the manuscript. ES collected data and played a leadership role in analyzing and interpreting data. AR planned the data collection effort and played a leadership role in analyzing and interpreting data. CC, NL, AS, JP, and JB played a leadership role in analyzing and interpreting data.

## Conflict of Interest Statement

The authors declare that the research was conducted in the absence of any commercial or financial relationships that could be construed as a potential conflict of interest.
